# A Buffering Role of Perceived Social Support and Resilience between Caregiver Burden and Perceived Stress among Informal Caregivers of Dementia Elderly Patients

**DOI:** 10.1186/s12877-026-07351-8

**Published:** 2026-04-02

**Authors:** Saba Zahid Hayat, Muhammad Saad, Syeda Razia Bukhari, Naila Naz

**Affiliations:** 1 Department of Psychology, Bahria University, Islamabad, Pakistan; 2 Department of Social Sciences, Iqra University, Islamabad, Pakistan; 3 Department of Social Science, SZABIST, Islamabad, Pakistan; 4 Department of Professional Psychology, Bahria University, Islamabad, Pakistan

**Keywords:** Dementia, Caregivers of dementia, Alzheimer's, Elderly/old population Gerontology, Longterm Care, Ageing

## Abstract

**Background:**

The psychological health of caregivers of dementia patients is at risk due to excessive burden, stress, and emotional strain. The study aimed to investigate the relationship between caregiver burden, perceived stress, resilience, and perceived social support among informal caregivers of dementia patients.

**Method:**

A purposive and snowball sampling technique was used to recruit a sample of 101 adult informal caregivers (immediate family members) of dementia patients, ranging in age from 18 to 60 years. Data were collected using English versions of the Caregiver Burden Scale (Gerontologist 20:649-655, 1980), the Brief Resilience Scale (Int J Behav Med 15:194-200, 2008), the Perceived Stress Scale (J Health Soc Behav 24:385-396, 1983), and the Multidimensional Scale of Perceived Social Support (J Pers Assess 52:30-41, 1988). SPSS—27 and PROCESS Macro (Model 6) by Hayes (2022) were employed for data analysis.

**Result:**

The findings supported the hypothesized relationships among all tested variables. Serial mediation revealed that perceived social support and resilience partially mediated the relationship between caregiver burden and perceived stress. However, no significant interaction was found between perceived social support and resilience in predicting perceived stress. The results suggest that both perceived social support and resilience are important buffers between caregiver burden and perceived stress. Additionally, sociodemographic analyses indicated that the caregiver burden varied according to the type of caregiving responsibilities informal caregivers provided.

**Conclusion:**

The study expands existing caregiving models by testing the buffering roles of social support and resilience among Pakistan informal dementia caregivers. The study highlights that, alongside elderly patients, their informal caregivers also require psychological support and intervention. It emphasizes the importance of developing intervention-based programs to reduce excessive burden and perceived stress among informal caregivers of dementia patients in Pakistan.

## Introduction

### Background of study

Informal caregivers, constituting a significant portion of the population, provide regular care to close relations without occupational compensation. According to the World Health Organization around 349 million individuals globally provide informal care, often without adequate resources or institutional support [19, 48]. The burden experienced by these caregivers, particularly those caring for individuals with dementia, is a multifaceted phenomenon encompassing emotional, physical, and financial challenges, often leading to a decline in their overall well-being [10, 14]. Dementia is an incurable and severe degenerative disease that not only affects a patient but also profoundly impacts those closely associated with them [24]. Caregiving for individuals with dementia is recognized as a chronic stressor that negatively affects caregivers’ mental, behavioral, and physical health [16, 40].

The lives of caregivers are often disrupted, with constraints on social life and conflicts between caregiving responsibilities and occupational roles, further highlighting the pervasive impact of their duties [47]. Caregiver burden, therefore, encapsulates the multifaceted strain experience by individuals providing care to family members or loved ones over extended periods [29]. This manifests as a complex biopsychosocial response resulting from an imbalance between the demands of caregiving and the resources available to caregivers, such as time, social support, financial means, physical and emotional health, and access to formal care services [3]. This burden is further exacerbated by the multiple roles caregivers are often required to fulfill beyond their caregiving responsibilities [3]. Studies have concluded that caregiving of dementia patients is stressful and overburdening, regardless of the stage of disease, and can, in many instances, lead to depression [27, 46]. Research also highlights signs of post-traumatic stress disorder (PTSD) among the informal caregivers of dementia patients [45]. Furthermore, poor quality of life and suicidal ideation has been reported among in caregivers experiencing high level of caregiver burden [40].

In a collectivistic culture like Pakistan, “*duty to care*” for elders is deeply embedded in social and religious values, viewed as a requisite for fulfilling filial responsibilities [5]. This duty is linked to religious practices and filial piety, therefore, individuals who deviate from these expectations often regarded as disobedient or apathetic towards their families [51]. Additionally, a pervasive stigma associated with dementia in Pakistan contributed to limited awareness, increased dependency, and neglect for dementia patients, particularly those with Alzheimer’s disease [1]. Consequently, formal services for dementia patients, including specialized nursing homes and respite care facilities remain scarce [38]. In such communal systems, social support serves as a critical coping mechanism and protective factor for both patients and their caregivers [25]. Theoretical frameworks such as stress-coping model, resilience theory, and social support buffering hypothesis consistently emphasize the importance of social support and personal resources in mitigating caregiver’s stress and enhancing well-being [6, 25, 38]. The caregiver’s capacity to adapt to these demands is strongly associated with resilience, which can be defined as a dynamic process involving positive adaptation and effective coping in the face of adversity, trauma, or significant stresses [9, 31, 42].

The caregiver burden refers to the multidimensional strain experienced by individuals providing unpaid care to chronically ill family members, encompassing physical, emotional, and financial stressors [20, 28]. Gender differences in caregiver burden have also been observed, with women experiencing higher emotional burden and men reporting greater financial strain [21]. In Pakistan, research has shown that many children caring for parents with Alzheimer’s disease forgo marriage to avoid additional responsibilities, consequently experiencing severe depression, anxiety, psychosomatic symptoms, and social isolation [2]. Caregiver burden refers to the multidimensional strain (emotional, physical, and financial) experienced by informal caregivers, as conceptualized by Zarit et al. [50].

Perceived stress, defined as an individual’s subjective appraisal of life events as unpredictable, uncontrollable, or overwhelming, significantly affects both mental and physical health [4]. It is influenced by factors such as life experiences, coping resources, and available support systems [8]. Studies show that perceived stress is closely linked to caregiver burden [44]. According to Lazarus and Folkman’s transactional stress model, stress arises when perceived demands exceed available coping resources [15]. Thus, caregiver’s appraisals of their caregiving responsibilities directly predict their stress levels. Recent studies in Pakistan confirm these patterns, reporting a positive relationship between caregiver burden and perceived stress, while both were inversely associated with perceived social support and resilience [21]. Consistently, international research has shown that dementia caregivers experience significantly higher perceived stress than control groups highlighting the widespread psychological impact of caregiving across cultures [38]. Caregivers who have negative beliefs about their ability to cope and those who feel trapped in their role have higher rates of morbidity and depressive symptoms [44]. These findings collectively underscore the urgent need for culturally sensitive interventions that target perceived stress to alleviate caregiver burden and improve the well-being of dementia caregivers in Pakistan [1].

Social Support is the key protective factor for informal caregivers. The classic *social support buffering hypothesis* holds that social support can have a direct salutary effect and mitigate (buffer) the adverse impact of stress [23, 35]. Cobb conceptualized social support as an informal, health-protective resource that takes various forms: emotional support, offering empathy, care, and encouragement,instrumental support, providing tangible assistance with practical tasks; and informational support, sharing advice, knowledge, or guidance [12, 34, 36]. However, among these, it is often the perceived availability of such support that exerts the most significant influence on caregivers’ psychological well-being.

Numerous studies indicate that caregivers with a high level of available social support report better mental health outcomes. In fact, *subjective perceptions* of available support tend to predict health outcomes more effectively than objective measures of received support [23, 26, 33, 40]. Specifically, for informal dementia caregivers, emotional and positive social interaction are associated with lower levels of burden and perceived stress [35]. For example, Ong et al. [35] found that emotional and affective support substantially reduces caregivers’ psychological burden. In collectivistic societies like Pakistan, families typically rely on informal support networks. Strong and cohesive networks can buffer stress, and reduce caregiver burden, whereas weak, fragmented, or overwhelmed networks can exacerbate caregiver strain, highlighting the crucial role of social support in mitigating the adverse psychological effects of caregiving [35].

Apart from social support serving as an external protective factor, resilience functions as an internal psychological resource that shields individuals during periods of adversity and emotional distress [30]. Contemporary theories conceptualize resilience as a dynamic process through which individuals maintain or restore their psychological well-being in response to stress [18]. Enhanced resilience has been consistently associated with reduced emotional distress among caregivers [41]. Key components that promote resilience include coping skills, optimism, and external resources such as social support [32]. Evidence suggests that caregivers with higher levels of resilience experience a reduced caregiving burden [35]. Similarly, caregivers who adopt problem-focused coping strategies and actively engage with supportive family or community networks tend to report better psychological outcomes. In South Asian cultural contexts, religious faith and deep-rooted spirituality play a significant role in reinforcing resilience [22]. This aligns with Bronfenbrenner’s ecological framework which emphasizes the influence of social norms, religious values, and family systems in shaping caregiving experiences. For example, strong filial piety and extended family structures in collectivistic societies like Pakistan provide foundation to emotional and practical support but can simultaneously heighten expectations and caregiving demands [51].

**Fig. 1:**
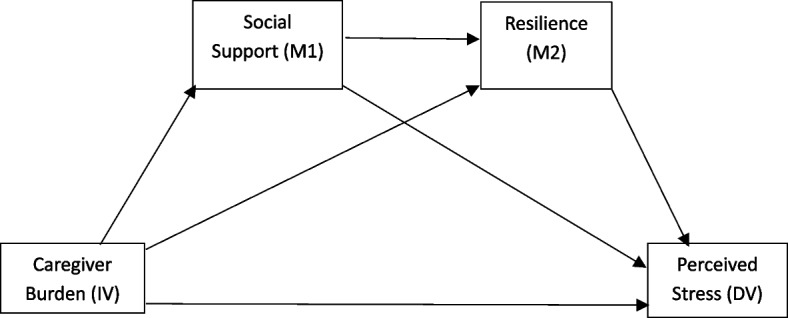
Conceptual diagram

Together these theoretical frameworks predict that higher perceived social support and resilience buffer the adverse effects of caregiving burden on perceived stress [35]. Empirical findings consistently demonstrate that caregivers with robust social support networks and strong coping resources report lower stress, and burnout, psychological distress [40]. Conversely, insufficient support and low resilience can exacerbate the negative psychological consequences of caregiving burden [30]. Within Pakistan’s context of limited formal dementia care services, understanding these dynamics is crucial for developing culturally appropriate interventions. Strengthening family and community support systems while fostering caregiver resilience may protect informal caregivers from the chronic burden and stress. The present study uniquely addresses a gap in South Asian research by testing a serial mediation model (social support→ resilience) in Pakistani dementia caregivers, an underexplored cultural context. The present study hypothesize that perceived social support and resilience will mediate the relationship between caregiver burden and perceived stress (Fig. 1). Additionally, the study will explore the influence of sociodemographic factors on caregiver burden and perceived stress.

## Methods

### Study design

To assess the relationship among the study variables, a correlational research design was employed using a cross-sectional survey method. Data was collected from the two adjoining cities of Pakistan: Islamabad and Rawalpindi. Ethical approval for the study was obtained from the relevant institutional review board. Purposive sampling and snowball sampling techniques were used to acquire data of the informal caregivers of dementia patients. To access the data, specialized clinics, and in particular Alzheimer’s Society Pakistan (A non-profit organization) served as a platform to contact the caregivers of dementia patients across the cities. 

### Participants

Participants were recruited through Alzheimer’s Pakistan (Rawalpindi chapter), specialized dementia clinics, and caregiver support groups. Snowball sampling was also used to reach hidden caregiver populations. The recruitment approach, while non-random, reflects typical access pathways in Pakistan. The age range of the informal caregivers was kept open ranging from 18–60 years of age. However, the participant had to be an informal caregiver of dementia patients providing significant caregiving support to the patient at home either part-time or full-time. In total, data was gathered from *N* = 120 participants, however, 5 declined participation and 14 surveys were incomplete thus finalizing data at *N* = 101. Informed written consent was obtained from all participants prior to data collection. The final sample size (*N* = 101) was determined based on feasibility and consistency with prior mediation studies in caregiving research (e.g., [17, 35]. According to Cohen’s [53] power analysis, this sample was adequate to detect medium effect sizes with 80% power.

### Procedure

Participants were approached through referrals from physicians as well as through Alzheimer’s Pakistan community programs. Upon signing the consent letter and participants were asked to fill out questionnaire-based scales along with sociodemographic information. Sociodemographic information was first obtained from the participants, including their marital status, sex, education level and self-rated financial status. Additionally, participants were asked to report their relationship with the dementia patient and the type of caregiving service they provided such as part-time or full-time care to dementia patients at home. Participants were also asked to report if they have any support to care for the dementia patient. Caregiving intensity ranged from 0–24 h/day, and caregiving roles were classified as physical help, financial help, or full responsibility (see Table 1). Whether caregiving was shared as a sole was also documented. Ethical considerations were given utmost importance during data collection.

**Table 1:** Sociodemographic characteristics and the distribution of caregiver burden and perceived stress among informal caregivers of dementia patients (*N* = 101)

Baseline Characteristics	*n* (%)	Caregiver Burden (*M* + *SD*)	*F/t*	*p*	Perceived Stress (*M* + *SD*)	*F/t*	*p*
Gender			1.05	0.30		−1.19	0.24
Male	51 (50%)	46.49 ± 15.70			21.03 ± 4.50		
Female	50 (50%)	43.24 ± 15.47			22.08 ± 4.37		
Age			0.53	0.46		2.51	0.11
18–35 (Adults)	34 (33.7%)	46.03 ± 16.95			21.68 ± 3.54		
36- 60 (Old Adults)	67 (66.3%)	44.30 ± 14.96			21.49 ± 4.86		
Job Status			1.00	0.32		0.42	0.51
Employed	54 (53.5%)	46.35 ± 13.36			21.61 ± 4.15		
Unemployed	56 (46.5%)	43.19 ± 17.81			21.49 ± 4.81		
Family Type			0.53	0.46		0.36	0.54
Nuclear	33 (32.7%)	42.42 ± 16.62			21.30 ± 4.49		
Joint	68 (67.3%)	46.07 ± 15.05			21.68 ± 4.45		
Hours of Caregiving			.002	0.88		1.48	0.22
0–12 h. a day	59 (60%)	43.57 ± 15.30			21.43 ± 3.99		
12–24 h. a day	40 (39.50%)	46.51 ± 15.97			21.71 ± 5.00		
RoCG			4.45	0.01		0.77	0.46
Physical help	21 (20.8%)	36.24 + 19.06			20.48 + 5.39		
Financial Help	20 (19.8%)	45.80 + 13.96			21.80 + 5.04		
All responsibilities	60 (59.4%)	47.60 + 15.59			21.55 + 4.44		

*PwD* Patient with Dementia, *RoCG* Responsibility of Caregiver

### Measures

#### Sociodemographic questionnaire

A sociodemographic questionnaire was used to collect information on participants’ marital status, sex, education level, self-rated financial status, relationship with dementia patient, and type of caregiving provided (part/full-time). Participants were also asked whether they had additional support in providing care.

#### Caregiver burden scale

The Zarit Burden Interview (ZBI) was used to assess caregiver burden [50]. The 22 items in the English version of ZBI are scored on a 5-point Likert scale, with 0 representing never and 4 representing almost usually. The total score falls between 0 and 88. With questions like "*do you feel or do you wish…*" and a Likert scale with a range of 0–4 (0 = *never*, 1 = *rarely*, 2 = *sometimes*, 3 = *rather frequently*, and 4 = *virtually always*), each item evaluates a participant's subjective load. ZBI has a total score range of 0 to 88, where higher values denote more stress. A heavy burden was defined as a score of 17 or higher [50]. Cronbach's *α*, as determined by the reliability analysis, was 0.90.

#### The brief resilience scale

The Brief Resilience Scale (BRS) was created to gauge people's perceived ability to recover or bounce back from adversity [43]. The scale was developed to assess a unitary construct of resilience and contains both positively and negatively phrased items. The brief resilience scale consists of 6 items with 3 items of negative scoring. The BRS is highly recommended due to its high reliability, Cronbach’s *α* = 0.71. The items 1 to 6 are marked on the Likert scale of 1–5. The Likert scale assigns a score on following range: 1 = *strongly disagree*, 2 = *disagree*, 3 = *neutral*, 4 = *agree*, and 5 = *strongly agree* for items 1, 3, and 5. The Likert scale for items 2, 4, and 6 is as follows:5 = *strongly disagree*, 4 = *disagree*, 3 = *neutral*, 2 = *agree*, and 1 = *strongly agree*. The responses are added and range between 6 and 30, divided by the total number of questions answered. A low score of BRS denotes low resilience, whereas high score shows exceptional resilience.

#### The perceived stress scale

The perceived stress scale consists of a total of 10 items. This scale was developed in 1983 but due to its high reliability and validity it is still used by researchers [13]. The PSS −10 has demonstrated good reliability (*a* = 0.78 in this study). The Likert scale ranging from 0–4 with following representation; 0 = never, 1 = almost never, 2 = occasionally, 3 = often, and 4 = very often, is used to rate the ten items. After reversing the scores for items 4, 5, 7, and 8, the total sum of the items is calculated to determine the level of stress. Scores range 0–40, with higher scores reflecting greater perceived stress. Scores above 20 typically indicate elevated stress levels.

### Multidimensional scale for perceived social support

The 12-item MSPSS measures three types of perceived social support: family, friends, and significant others. This measure is graded using a Likert scale, where 1 = *very strongly disagree*, 2 = *strongly disagree*, 3 = *mildly disagree*, 4 = *neutral*, 5 = *mildly agree*, 6 = *strongly agree*, and 7 = *very strongly agree*. Items 4, 6, 7, and 9 are for friends’ support, items 3, 4, 8, and 11 are for family support and items 1, 2, 5 and 10 represent significant other support. MSPSS carries good internal reliability: total scale *a* = 0.88; family support *a* = 0.87; friends’ support *a* = 0.85 and significant other support *a* = 0.91 [52].

### Statistical analysis

For assessing sociodemographic traits and comparing the distribution of caregiver burden, respectively, descriptive analysis, independent t-test, and one-way analysis of variance (ANOVA) were computed. Pearson Correlation analyses were carried out to evaluate the study variables' correlation. PROCESS macro for SPSS was used to investigate the serial multiple mediation model (Model 6) (Hayes 2013). 5000 bootstrapping re-samples were used to determine the bias-correlated 95% confidence interval (CI). For all statistical analyses, SPSS 27.0 was used. The study variables were standardized, and covariates (age, sex, marital status, education, and self-rated financial position) were controlled for statistical significance.

## Results

### Sociodemographic characteristics

To assess differences in caregiver burden and perceived stress based on various sociodemographic characteristics, One-way analyses of variance (ANOVA) and independent samples t-tests were used. The descriptive statistics, test values, and significance levels are presented in Table 1.

The analysis revealed that no gender differences were found for caregiver burden, *t* (99) = 1.05, *p* = 0.30, or perceived stress, *t* (99) = −1.19, *p* = 0.24. Similarly, no significant differences were observed between younger adults (18–35 years) and older adults (36–60) for caregiver burden, *t* (99) = 0.53,* p* = 0.46, or perceived stress *t* (99) = 2.51, *p* = 0.11. Caregiver burden did not differ significantly by job status, *t* (99) = 1.00, *p* = 0.32, or by family type (nuclear vs. joint),* t* (99) = 0.53,* p* = 0.46. Likewise, hours of caregiving per day (0–12 vs. 12–24 h) were not associated with significant differences in either caregiver burden,* t* (97) = 0.02,* p* = 0.88, or perceived stress,* t* (97) = 1.48, *p* = 0.22.

A significant effect was found for the role of caregiving on caregiver burden, *F* (2, 98) = 4.45, *p* = 0.01. Post hoc comparisons indicated that caregivers responsible for all caregiving responsibilities (*M* = 47.60, *SD* = 15.59) reported higher burden compared to those providing only physical help (*M* = 36.24, *SD* = 19.06). No significant group differences were observed in perceived stress across caregiving roles,* F* (2, 98) = 0.77, *p* = 0.46.

### Bivariate correlations among all the variables

The associations between caregiver burden, resilience, perceived stress, and perceived social support were investigated using a Pearson product-moment correlation analysis. Table 2 demonstrates the negative correlation between caregiver burden and perceived social support (*r* = −0.46, *p* < 0.01), resilience (*r* = −0.49, *p* < 0.01), and its subscales, such as intimate support (*r* = −0.38, *p* < 0.01). On the other hand, subjective stress and caregiver burden were positively connected. (*r* = 0.64, *p* < 0.01). Resilience was negatively correlated with perceived stress (*r* = −0.46, *p* < 0.01) and positively correlated with perceived social support (*r* = 0.24, *p* < 0.05), intimate support (*r* = 0.25, *p* < 0.05), but not significantly with family support (*r* = 0.14, *p* > 0.05) or friends’ support (*r* = 0.17, *p* > 0.05). Perceived stress was negatively associated with perceived social support (*r* = −0.35, *p* < 0.01), intimate support (*r* = −0.41, *p* < 0.01), family support (*r* = −0.39, *p* < 0.01), and friends’ support (*r* = −0.39, *p* < 0.01). Strong positive intercorrelations were also observed among the three perceived social support subscales ranging from *r* = 0.39 to *r* = 0.84 (*p* < 0.01).

**Table 2:** Pearson correlation table to assess the relationship between caregiver burden, social support, perceived stress and resilience (*N* = 101)

Variables	1	2	3	4	5	6	7
1. Caregiver Burden	—						
2. Resilience	-.49**	—					
3. Perceived Stress	.64**	-.46**	—				
4. Perceived Social Support	-.46**	.24*	-.35**	—			
5. PSS – Intimate	-.37**	.25*	-.41**	.84**	—		
6. PSS– Family	-.36**	.14	-.39**	.83**	.61**	—	
7. PSS– Friends	-.38**	.17	-.39**	.76**	.39**	.48**	—

*PSS* Perceived Social Support

^*^p. <.05

^**^ *p* <.01

### Multiple regression analysis

The results indicated that the caregiver burden significantly predicted perceived social support, *F* (1,99) = 27.18, *p* < 0.001, *R*^*2*^ = 0.215, resilience, *F* (1, 99) = 16.11, *p* < 0.001, *R*^*2*^ = 0.247, and perceived stress, *F* (1, 99) = 29.50, *p* < 0.001, *R*^*2*^ = 0.477. Specifically, the caregiver burden explained 21.5% of the variance in perceived social support (*R* = 0.464), 24.7% of the variance in resilience (*R* = 0.497), and 47.7% of the variance in perceived stress (*R* = 0.691). Additionally, the total effect of caregiver burden on these outcome variables was also significant, *F* (1, 99) = 68.74, *p* < 0.001, accounting for 41% of the combined variance (*R* = 0.640) as shown in Table 3. The higher MSE value observed in the regression model for perceived social support reflects the greater variance in social support scores relative to other outcomes measures and is consistent with the distribution of the data.

**Table 3:** Multiple regression analysis predicting perceived social support, perceived stress and resilience (*N* = 101)

**Outcome**		**Caregiver Burden**			
	***R***	***R***^***2***^	***MSE***	***F***	***P***
Perceived Social Support	.464	.215**	168.56	27.18	<.01
Resilience	.497	.247**	10.19	16.11	<.01
Perceived Stress	.691	.477**	10.67	29.50	<.01
Total effect	.640	.410**	11.80	68.74	<.01

95% confidence intervals

^*^p. <.05

^**^ *p* <.01

### Serial mediation analysis (Model 6)

A serial multiple mediation analysis was conducted using PROCESS macro (Model 6; [11]) to examine whether perceived social support and resilience serially mediate the relationship between caregiver burden and perceived stress. The analysis used 5,000 bootstrap samples with 95% percentile confidence intervals.

**Fig. 2:**
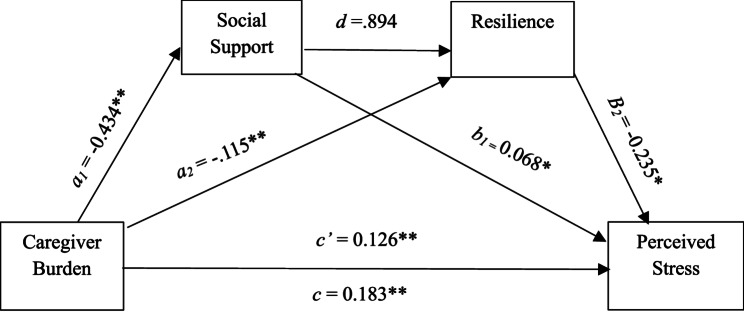
Statistical Diagram of serial mediation analysis among variables

As shown in Table 4, results indicated that caregiver burden negatively predicted perceived social support (B = −0.43, SE = 0.08, t = −5.21, *p* < 0.001) and resilience (B = −0.12, SE = 0.02, t = −4.96, *p* < 0.001). In predicting perceived stress, caregiver burden (B = 0.13, SE = 0.03, t = 4.76, *p* < 0.001), social support (B = −0.07, SE = 0.03, t = −2.68, *p* = 0.009), and resilience (B = −0.24, SE = 0.10, t = −2.27, *p* = 0.025) all demonstrated significant direct effects. The total effect of caregiver burden on perceived stress was also significant (B = 0.18, SE = 0.02, t = 8.29, *p* < 0.001) (demonstrated in Fig. 2).

**Table 4:** Regression coefficients of Serial Mediation Model between Caregiver Burden, Perceived Social Support, Resilience and Perceived Stress (N=101)

Outcome	Predictor	*B*	*SE*	*t*	*P*	*LLCI*	*ULCI*	*β*
Perceived Social	Constant	68.94	3.95	17.44	<.001	61.10	76.79	-
Support	Caregiver Burden	-0.434	0.083	-5.21	<.001	-.599	-0.269	-0.464**
Resilience	Constant	23.23	1.96	11.84	<.001	19.34	27.12	-
	Caregiver burden	-0.115	0.023	-4.96	<.001	-0.161	-0.069	-0.491**
	P-Social Support	0.003	0.025	0.13	.894	-0.046	0.052	0.013
Perceived Stress	Constant	23.53	3.13	7.52	<.001	17.32	29.74	-
	Caregiver burden	0.126	0.026	4.76	<.001	0.074	0.179	0.442**
	P-Social Support	-0.068	0.025	-2.68	.009	-0.118	-0.018	-0.222*
	Resilience	-0.235	0.103	-2.27	.025	-0.440	-0.030	-0.192*
Perceived Stress	Constant	13.36	1.05	12.77	<.001	11.28	15.43	-
(Total effect)	Caregiver burden	0.183	0.022	8.29	<.001	0.139	0.226	0.640**

*p. <.05, ** *p* <.01; P - perceived

A serial mediation analysis revealed that caregiver burden had a significant total effect on perceived stress (*B* = 0.183, *p* < 0.001). The direct effect remained significant after accounting for mediators (*B* = 0.126, *p* < 0.001), indicating partial mediation. Total indirect effect was significant (*B* = 0.057, *p* < 0.001). Specifically, caregiver burden influenced perceived stress indirectly through perceived social support (*B* = 0.029) and resilience (*B* = 0.026). However, the serial indirect path through both perceived social support and resilience was non-significant (*B* = 0.000). These findings support partial mediation.

## Discussion

The study investigated the relationship between caregiver burden and perceived stress among the informal caregivers of dementia patients, while exploring the serial mediating roles of perceived social support and resilience. Data was collected from a sample of *N* = 101 informal caregivers residing in Islamabad and Rawalpindi, Pakistan. A purposive and snowball sampling technique was employed for recruitment, with assistance of NGOs such as Alzheimer’s Pakistan (Rawalpindi Chapter), which played a pivotal role in facilitating access to the target population.

As hypothesized, results revealed that caregiver burden was significantly associated with higher perceived stress, suggesting that caregivers reporting greater burden also tended to report more stress (as shown in Table 3). This finding is consistent with previous literature, which has highlighted the substantial emotional, physical, and psychological strain experienced by informal dementia caregivers [7]. The caregiver burden had significant negative relationship with perceived social support and resilience (Table 3), aligning with previous literature that highlights how increased caregiving demands often erode available social support and diminish personal coping capacity [17, 49].

The mediation analysis revealed a partial serial mediation effect. Both perceived social support and resilience independently mediated the relationship between caregiver burden and perceived stress. Specifically, the higher caregiver burden was associated with reduced perceived social support, which in turn predicted higher perceived stress. This indirect effect via perceived social support alone is statistically significant (Fig. 2). Similarly, caregiver burden also negatively predicted resilience, which subsequently predicted lower perceived stress, confirming mediating role of resilience. These findings corroborate with prior evidence suggesting that social support and resilience are critical protective factors against psychological distress in caregiving populations [17, 49].

On the contrary, the serial indirect pathway through both perceived social support and resilience in sequence was not statistically significant (Table 4). This indicates that while both mediators exert individual influence on perceived stress, there is no evidence for a causally linked pathway whereby caregiver burden reduces perceived social support, which then impairs resilience and subsequently increases perceived stress (Table 5). This finding suggests that in the Pakistani caregiving context, perception of having social support and internal coping mechanism as resilience function as parallel, rather than sequential, buffers against caregiver perceived stress. The absence of a direct mediating effect between perceived social support and resilience necessitates a critical examination of the socio-cultural context of informal caregiving in Pakistan, where familial obligations and collectivistic values often shape caregiving experiences. It is plausible that the availability of social support, while acknowledged and potentially utilized, does not automatically translate into enhanced resilience among caregivers [35].

**Table 5:** Path analysis of serial mediation model between caregiver burden, perceived social support, resilience and perceived stress (*N* = 101)

Paths	*B*	*Boot SE*	*95% CI*		*P*
			***(LLCI, ULCI)***		
Total Effect	0.183	0.022	0.139	0.226	Significant
Direct Effect	0.126	0.026	0.074	0.179	Significant
Total Indirect Effect	0.057	0.015	0.027	0.088	Significant
*Ind 1* (Caregiver burden → P-Social Support → Perceived Stress)	0.029	0.010	0.010	0.052	Significant
*Ind 2* (Caregiver burden → Resilience → Perceived Stress)	0.026	0.012	0.005	0.052	Significant
*Ind 3* (Caregiver burden → P-Social Support → Resilience → Perceived Stress)	0.000	0.003	−0.005	0.008	Non-significant
Conclusion				Partial Mediation	

*P* Perceived

Importantly, the direct effect of caregiver burden on perceived stress remained significant after accounting for both mediators (Table 5). This implies that while perceived social support and resilience partially explain the relationship, other unmeasured factors (such as financial hardship, access to healthcare resources, and cultural stigma) may also play an important role in shaping caregivers’ stress experiences. The results align with established theoretical frameworks such as the stress-process model [37], which posits that caregiver burden contributes to psychological distress through both direct effects and indirect effects mediated by coping resources. The findings also extend prior research in similar contexts by demonstrating the importance of both interpersonal (social support) and intrapersonal (resilience) resources in mitigating caregiver stress [17, 49].

Furthermore, the analysis of sociodemographic differences revealed that caregiver burden levels differed significantly based on caregiving role. As shown in Table 1, caregivers taking full responsibility for all caregiving tasks reported a greater burden than those providing only physical or financial support. These differences highlight the heterogeneity within the caregiver population suggest that specific subgroups may be at greater risk of experiencing high burden [21]. Furthermore, there was no difference found in gender across the variables. Similarly, the test revealed that perceived stress did not differ significantly across any of the assessed sociodemographic characteristics. This suggests that while the experience of burden varies by demographic factors, the psychological experience of stress appears to me to be a more universal outcome of caregiving strain, predominantly influenced by psychosocial factors such as social support and resilience [38]. In collectivist societies like Pakistan, where caregiving is often viewed as a familial obligation, stress may emerge independently of social position or demographic background once burden reaches a threshold. It can be analyzed that sociodemographic variables act as a contextual risk factor that influences levels of caregiver burden, which then exert their effects on perceived stress through psychosocial mediators.

Analyses controlled for covariates identified in prior research, including age, sex, marital status, education, and financial status. Although exploratory interaction terms (e.g., gender x caregiving role) were tested, the modest sample size limited statistical power; results were non-significant and are reported in limitations.

The study limitations suggest that cross-sectional design restricts the ability to infer causal relationships among caregiver demands, perceived social support, resilience, and perceived stress. The perceived social support measures subjective support rather than objective support to infer the existing support for caregivers. Additional self-report measures introduce the possibility of recall bias and social desirability effects. Modest sample size (*N* = 101) due to availability of special sample population may have limited statistical power to detect the subtler serial mediation. Unmeasured confounders, such as severity of dementia, financial strain, and access to healthcare, were not assessed. Objective measures of caregiving intensity are also absent.

Despite limitations, the study carries both theoretical and practical implications. Theoretically, it supports stress-process models [37] by confirming both direct and indirect effects of caregiving demands on distress. Practically, results suggest the need for targeted interventions prioritizing employed caregivers and those providing full-time, comprehensive care. These caregivers represent vulnerable subgroups likely to experience elevated burden and stress, warranting workplace policies and flexible caregiving resources and psychological assistance by the governing bodies. Additionally, universal interventions aimed at enhancing social support networks and promoting resilience-building strategies could benefit all caregivers, regardless of demographic characteristics. Non-profit organizations such as Alzheimer’s Pakistan should be given public platform to create awareness to reduce caregiving stigma. Future studies can measure objective social support through exploring the availability of social support and use that knowledge build intervention across cities. Future Studies may include more mediators such as stigma, financial strain and healthcare accessibility, especially in the under-developed countries.

In conclusion, this study underscores the profound impact of caregiver burden on perceived stress among informal caregivers of dementia patients in Pakistan. Both perceived social support and resilience emerged as independent protective factors, although no empirical evidence was found for a sequential relationship between them. The persistence of the direct effect of caregiver burden on perceived stress, even after accounting for these psychosocial resources, highlights the multifaceted nature of caregiving stress within collectivistic societies. These findings emphasize the need for culturally adapted, resource-sensitive interventions that address not only caregiver’s psychosocial resources but also the broader sociocultural, financial, and healthcare challenges that shape their caregiving experience. The study contributes to the growing literature on informal caregiving in South Asia and offers valuable directions for future research and intervention development.

## Data Availability

The datasets used and/or analyzed during the current study are available from the corresponding author on reasonable request.

## References

[1] Ali AM, Alkhamees AA, Hallit S, Al-Dwaikat T, Khatatbeh H, Al-Dossary SA. The Depression Anxiety Stress Scale 8: investigating its cutoff scores in relevance to loneliness and burnout among dementia family caregivers. Sci Rep. 2024;14(1):13075. 10.1038/s41598-024-60127-1. 38844485 10.1038/s41598-024-60127-1 PMC11156668

[2] Ali S, Bokharey IZ. Maladaptive cognitions and physical health of the caregivers of dementia: an interpretative phenomenological analysis. Int J Qual Stud Health Well-being. 2015;10(1):28980. 10.3402/qhw.v10.28980. 26384522 10.3402/qhw.v10.28980 PMC4575415

[3] Alnazly EK, Samara NA. The burdens on caregivers of patients above 65 years old receiving hemodialysis: a qualitative study. Health Care Curr Rev. 2014;2(1):118. 10.4172/hccr.1000118.

[4] Ameri F, Rahmani H, Mirhosseini S, Basirinezhad MH, Saeedi MM, Ebrahimi H. Exploring caregiver burden in Alzheimer’s disease: the predictive role of psychological distress. Open Public Health J. 2024. 10.2174/0118749445327572240916091208.

[5] Awan K, Ahmad N, Naveed RT, Scholz M, Adnan M, Han H. The impact of work–family enrichment on subjective career success through job engagement: a case of banking sector. Sustainability. 2021;13(16):8872. 10.3390/su13168872.

[6] Bilal A, Saeed MA, Yousafzai T. Elderly care in the time of coronavirus: perceptions and experiences of care home staff in Pakistan. Int J Geriatr Psychiatry. 2020;35(12):1442. 10.1002/gps.5386. 32748399 10.1002/gps.5386 PMC7436612

[7] Bolin JH. Hayes AF. (2013). Introduction to Mediation, Moderation, and conditional process analysis: A regression-based approach. New York, NY: The Guilford Press. J Educ Meas. 2014;51:335-7. 10.1111/jedm.12050.

[8] Can YS, Iles‐Smith H, Chalabianloo N, Ekiz D, Fernández‐Álvarez J, Repetto C, et al. How to relax in stressful situations: a smart stress reduction system. Healthcare. 2020;8(2):100. 10.3390/healthcare8020100. 32316370 10.3390/healthcare8020100 PMC7349817

[9] Chang Y, Yang C, Hsieh S. Social support enhances the mediating effect of psychological resilience on the relationship between life satisfaction and depressive symptom severity. Sci Rep. 2023;13(1):4818. 10.1038/s41598-023-31863-7. 36964160 10.1038/s41598-023-31863-7 PMC10036971

[10] Choi JY, Lee SH, Yu S. Exploring factors influencing caregiver burden: a systematic review of family caregivers of older adults with chronic illness in local communities [Review of Exploring Factors Influencing Caregiver Burden: A Systematic Review of Family Caregivers of Older Adults with Chronic Illness in Local Communities]. Healthcare. 2024;12(10):1002. 10.3390/healthcare12101002. 38786412 10.3390/healthcare12101002 PMC11121359

[11] Cohen J. Statistical Power Analysis. Curr Dir Psychol Sci. 1992;1(3):98-101. 10.1111/1467-8721.ep10768783.

[12] Cohen S, Wills TA. Stress, social support, and the buffering hypothesis. Psychol Bull. 1985;98(2):310. 10.1037/0033-2909.98.2.310. 3901065

[13] Cohen S, Kamarck T, Mermelstein R. A global measure of perceived stress. J Health Soc Behav. 1983;24(4):385–96. 10.2307/2136404. 6668417

[14] Edwards VJ, Bouldin ED, Taylor CA, Olivari B, McGuire LC. Characteristics and health status of informal unpaid caregivers — 44 states, District of Columbia, and Puerto Rico, 2015–2017. MMWR Morb Mortal Wkly Rep. 2020;69(7):183. 10.15585/mmwr.mm6907a2. 32078592 10.15585/mmwr.mm6907a2 PMC7043388

[15] Ferrer I, Castro JF, Edo S, Rovira T. The influence of the primary and secondary appraisals, and of the big five personality traits, on the choice of coping strategies: a study based on daily stress. Stud Psychol. 2021;63(3):266. 10.31577/sp.2021.03.826.

[16] Feyisa HL, Endalew AM, Tibebu K, Mossie D. The economic cost and burden of informal caregiving for the inpatient: the case of Lemlem Karl Hospital, Maichew, Ethiopia. Int J Econ Energy Environ. 2020;5(2):14. 10.11648/j.ijeee.20200502.11.

[17] Fiorentino DF, Chi SCC, Chua HC, See SCC, Lim SH, Chan YH. Resilience and burden in caregivers of older adults: moderating and mediating effects of perceived social support. BMC Psychiatry. 2018;18(1):161. 10.1186/s12888-018-1616-z. 29385985 10.1186/s12888-018-1616-z PMC5793423

[18] Fu H, Wang N. The current state of research on resilience, and its relationship to education. Lecture Notes in Education Psychology and Public Media. 2023;2(1):910. 10.54254/2753-7048/2/2022584.

[19] Gao C, Chapagain M, Scullin C. Global estimates of informal caregiving and associated factors: a systematic review. Int J Environ Res Public Health. 2022;19(4):2152.35206336

[20] Graessel E, Berth H, Lichte T, Grau H. Subjective caregiver burden: validity of the 10-item short version of the Burden Scale for Family Caregivers BSFC-s. BMC Geriatr. 2014;14(1):23. 10.1186/1471-2318-14-23. 24555474 10.1186/1471-2318-14-23 PMC3942019

[21] Gümüşkaya O, Şen S, Işık I, Ayaz V, Özkan HA, Wilson R. Urban share of the “burden”: impact of a support organisation on caregiver burden of people affected by dementia. Perspect Psychiatr Care. 2023;2023(1):2706698. 10.1155/2023/2706698.

[22] Guribye E, Sandal GM, Oppedal B. Communal proactive coping strategies among Tamil refugees in Norway: a case study in a naturalistic setting. Int J Ment Health Syst. 2011;5(1):9. 10.1186/1752-4458-5-9. 21521494 10.1186/1752-4458-5-9 PMC3096987

[23] Irfan B, Irfan O, Ansari A, Qidwai W, Nanji K. Impact of caregiving on various aspects of the lives of caregivers. Cureus. 2017;9(5):e1213. 10.7759/cureus.1213. 28589062 10.7759/cureus.1213 PMC5453737

[24] Kahriman F, Zaybak A. Caregiver burden and perceived social support among caregivers of patients with cancer. Asian Pac J Cancer Prev. 2015;16(8):3313. 10.7314/apjcp.2015.16.8.3313. 25921137 10.7314/apjcp.2015.16.8.3313

[25] Knapińska Z, Mulawka J. Patient-tailored dementia diagnosis with CNN-based brain MRI classification. Appl Sci. 2025;15(9):4652. 10.3390/app15094652.

[26] Kua EH. Elderly people with mental illness in South-East Asia: rethinking a model of care. Int Psychiatry. 2010;7(2):34. 10.1192/s1749367600005701. 31508029 PMC6734966

[27] Lai DWL, Thomson C. The impact of perceived adequacy of social support on caregiving burden of family caregivers. Fam Soc J Contemp Soc Serv. 2011;92(1):99. 10.1606/1044-3894.4063.

[28] Langa KM, Chernew ME, Kabeto MU, Herzog AR, Ofstedal MB, Willis RJ, et al. National estimates of the quantity and cost of informal caregiving for the elderly with dementia. J Gen Intern Med. 2001;16(11):770. 10.1111/j.1525-1497.2001.10123.x. 11722692 10.1111/j.1525-1497.2001.10123.x PMC1495283

[29] Lee J, Peterson N, Zarit SH. Building caregiver resilience: pitfalls and potential for interventions. Innov Aging. 2021;5:53. 10.1093/geroni/igab046.202.

[30] Liu Z, Heffernan C, Tan J. Caregiver burden: a concept analysis. Int J Nurs Sci. 2020;7(4):438. 10.1016/j.ijnss.2020.07.012. 33195757 10.1016/j.ijnss.2020.07.012 PMC7644552

[31] Manzari ZS, Rafiei H, Ghaderi MS, Abedi F, Mafi MH. Relationship between resilience and caregiver burden among home caregivers of COVID-19 patients. Home Healthc Now. 2023;41(1):42. 10.1097/nhh.0000000000001133. 36607209 10.1097/NHH.0000000000001133 PMC9812295

[32] Manzini CSS, Brígola AG, Pavarini SCI, Vale FAC. Factors associated with the resilience of family caregivers of persons with dementia: a systematic review [Review of Factors associated with the resilience of family caregivers of persons with dementia: a systematic review]. Rev Bras Geriatr Gerontol. 2016;19(4):703. 10.1590/1809-98232016019.150117.

[33] Mousavi SM, Naeini MJ, Sadeghian M, Yazdanirad S. The Relationship Between Different Dimensions of Occupational Stress and Resilience Levels in the Employees of an Oil Refinery. 2021. DOAJ (DOAJ: Directory of Open Access Journals). https://doaj.org/article/6b8e4160f4e94ff18f7c3cedc9a2caa0.

[34] Munoz-Bermejo L, Adsuar JC, Postigo-Mota S, Casado-Verdejo I, deMelo-Tavares CM, Garcia-Gordillo MA, et al. Relationship of perceived social support with mental health in older caregivers. Int J Environ Res Public Health. 2020;17(11):3886. 10.3390/ijerph17113886. 32486267 10.3390/ijerph17113886 PMC7312634

[35] Omodan BI. Psychological implication of student unrest on student leaders: a social support perspective. Heliyon. 2023;9(11):e22334. 10.2139/ssrn.4434720. 38053862 10.1016/j.heliyon.2023.e22334 PMC10694326

[36] Ong HL, Vaingankar JA, Abdin E, Sambasivam R, Fauziana R, Tan M, et al. Resilience and burden in caregivers of older adults: moderating and mediating effects of perceived social support. BMC Psychiatry. 2018;18(1):27. 10.1186/s12888-018-1616-z. 29385985 10.1186/s12888-018-1616-z PMC5793423

[37] Özbay F, Johnson DC, Dimoulas E, Morgan CA, Charney DS, Southwick SM. Social support and resilience to stress: from neurobiology to clinical practice. PubMed. 2007;4(5):35–40 https://pubmed.ncbi.nlm.nih.gov/20806028. PMC2921311 20806028

[38] Pearlin LI, Mullan JT, Semple SJ, Skaff MM. Caregiving and the stress process: an overview of concepts and their measures. Gerontologist. 1990;30(5):583–94. 10.1093/geront/30.5.583. 2276631 10.1093/geront/30.5.583

[39] Pinquart M, Sorenson S. Association of stressors and uplift of caregiving with caregiver burden and depressive mood: A meta-analysis. J Gerontol B Psychol Sci Soc Sci. 2003;58(2):P112-28. 10.1093/geronb/58.2.p112. 10.1093/geronb/58.2.p112 12646594

[40] Qadir F, Gulzar W, Haqqani S, Khalid A. A pilot study examining the awareness, attitude, and burden of informal caregivers of patients with dementia. Care Manag J. 2013;14(4):230. 10.1891/1521-0987.14.4.230. 24579270 10.1891/1521-0987.14.4.230

[41] Schulz R, Sherwood PR. Physical and mental health effects of family caregiving [Review of Physical and Mental Health Effects of Family Caregiving]. AJN Am J Nurs. 2008;108(9):23. 10.1097/01.naj.0000336406.45248.4c. 18797217 10.1097/01.NAJ.0000336406.45248.4c PMC2791523

[42] Sharma G, Yukhymenko–Lescroart MA. Life purpose as a predictor of resilience and persistence in college students during the COVID-19 pandemic. J Coll Stud Retention Res Theory Pract. 2022;26(2):334. 10.1177/15210251221076828.

[43] Sisto A, Vicinanza F, Campanozzi LL, Ricci G, Tartaglini D, Tambone V. Towards a transversal definition of psychological resilience: a literature review [Review of Towards a Transversal Definition of Psychological Resilience: A Literature Review]. Medicina (Kaunas). 2019;55(11):745. 10.3390/medicina55110745. 31744109 10.3390/medicina55110745 PMC6915594

[44] Smith BW, Dalen J, Wiggins K, Tooley E, Christopher P, Bernard J. The brief resilience scale: assessing the ability to bounce back. Int J Behav Med. 2008;15(3):194–200. 10.1080/10705500802222972. 18696313 10.1080/10705500802222972

[45] Sörensen S, Conwell Y. Issues in dementia caregiving: effects on mental and physical health, intervention strategies, and research needs. Am J Geriatr Psychiatry. 2011;19(6):491. 10.1097/jgp.0b013e31821c0e6e. 21502853 10.1097/JGP.0b013e31821c0e6e PMC3774150

[46] Theng B, Tran JT, Serag H, Raji M, Tzeng H, Shih M, et al. Understanding caregiver challenges: a comprehensive exploration of available resources to alleviate caregiving burdens. Cureus. 2023;15(8):e43052. 10.7759/cureus.43052. 37680399 10.7759/cureus.43052 PMC10480575

[47] Thrush A, Hyder AA. The neglected burden of caregiving in low- and middle-income countries [Review of The neglected burden of caregiving in low- and middle-income countries]. Disabil Health J. 2014;7(3):262. 10.1016/j.dhjo.2014.01.003. 24947567 10.1016/j.dhjo.2014.01.003

[48] Wang F, Irani E. Caregiving stressors and mental health among older dementia caregivers: the mediating role of life disruption. Innov Aging. 2022;6:587. 10.1093/geroni/igac059.2203.

[49] World Health Organization. Global status report on care provision: Informal caregivers and health systems response. Geneva: WHO Press; 2023.

[50] Yu M, Qiu T, Liu C, Zhang Y, Li Z. The mediating role of perceived social support between anxiety symptoms and life satisfaction in pregnant women: a cross-sectional study. Health Qual Life Outcomes. 2020;18(1):223. 10.1186/s12955-020-01479-w. 32650793 10.1186/s12955-020-01479-w PMC7348126

[51] Zarit SH, Reever KE, Bach-Peterson J. Relatives of the impaired elderly: correlates of feelings of burden. Gerontologist. 1980;20(6):649–55. 10.1093/geront/20.6.649. 7203086 10.1093/geront/20.6.649

[52] Zarzycki M, Seddon D, Bei E, Dekel R, Morrison V. How culture shapes informal caregiver motivations: a meta-ethnographic review [Review of How Culture Shapes Informal Caregiver Motivations: A Meta-Ethnographic Review]. Qual Health Res. 2022;32(10):1574. 10.1177/10497323221110356. 35737473 10.1177/10497323221110356 PMC9411702

[53] Zimet GD, Dahlem NW, Zimet SG, Farley GK. The multidimensional scale of perceived social support. J Pers Assess. 1988;52(1):30–41. 10.1207/s15327752jpa5201_2.

